# NOVEL APPROACHES AND DATA SOURCES TO INVESTIGATE LIFESPAN ASSOCIATIONS FROM EARLY EXPOSURES TO LATER LIFE HEALTH

**DOI:** 10.1093/geroni/igae098.2072

**Published:** 2024-12-31

**Authors:** Lewina Lee

**Affiliations:** Chair

## Abstract

Despite substantial evidence linking early adversity to elevated risks of later-life morbidity and mortality, recent findings on the often-poor agreement between retrospective and prospective measures of childhood maltreatment (Κs=.09-.83; Baldwin et al., 2019) have challenged these well-established associations, which were predominantly derived from retrospectively-assessed early exposures in longitudinal cohorts. As most longitudinal aging studies enrolled participants in their adulthood, prospective early-life data combined with long-term tracking of health outcomes are scarce, but necessary for rigorous causal inference regarding the nature and mechanisms by which early exposures shape life span health. This symposium showcases recent methodological developments and new data resources capable of addressing these scientific barriers and amplifying the scientific value of longitudinal aging studies. Ms. Dorame will illustrate a novel approach to acquiring prospective data on childhood familial and environmental exposures by linking three longitudinal cohorts to recently digitalized, 1900-1940 US Censuses in the Boston Early Adversity and Mortality Study (BEAMS). Dr. Marino will report on the prospective associations of childhood and midlife socioeconomic status and all-cause mortality risk using BEAMS Census-linked data. Dr. Casey will describe efforts to assess early-life environmental exposures and evaluate their associations with later-life brain health in the Health and Retirement Study. Dr. Arpawong will consider the extent to which cognitive abilities were retained from adolescence to old age in Project Talent, and the roles of education and gender therein. Finally, Dr. Warren will describe three new, large-scale data resources suitable for interdisciplinary research on the later-life sequelae of early-life exposures. Measurement, Statistics, and Research Design Interest Group Sponsored Symposium

